# Characterizing Natural Organic Matter Transformations by Microbial Communities in Terrestrial Subsurface Ecosystems: A Critical Review of Analytical Techniques and Challenges

**DOI:** 10.3389/fmicb.2022.864895

**Published:** 2022-05-04

**Authors:** Kristine Grace M. Cabugao, Sara Gushgari-Doyle, Stephany S. Chacon, Xiaoqin Wu, Amrita Bhattacharyya, Nicholas Bouskill, Romy Chakraborty

**Affiliations:** Department of Ecology, Climate and Ecosystem Sciences Division, Lawrence Berkeley National Laboratory, Berkeley, CA, United States

**Keywords:** microbiome, subsurface, capillary fringe, natural organic matter, soil carbon

## Abstract

Determining the mechanisms, traits, and pathways that regulate microbial transformation of natural organic matter (NOM) is critical to informing our understanding of the microbial impacts on the global carbon cycle. The capillary fringe of subsurface soils is a highly dynamic environment that remains poorly understood. Characterization of organo-mineral chemistry combined with a nuanced understanding of microbial community composition and function is necessary to understand microbial impacts on NOM speciation in the capillary fringe. We present a critical review of the popular analytical and omics techniques used for characterizing complex carbon transformation by microbial communities and focus on how complementary information obtained from the different techniques enable us to connect chemical signatures with microbial genes and pathways. This holistic approach offers a way forward for the comprehensive characterization of the formation, transformation, and mineralization of terrestrial NOM as influenced by microbial communities.

## Introduction

**Figure 1:**
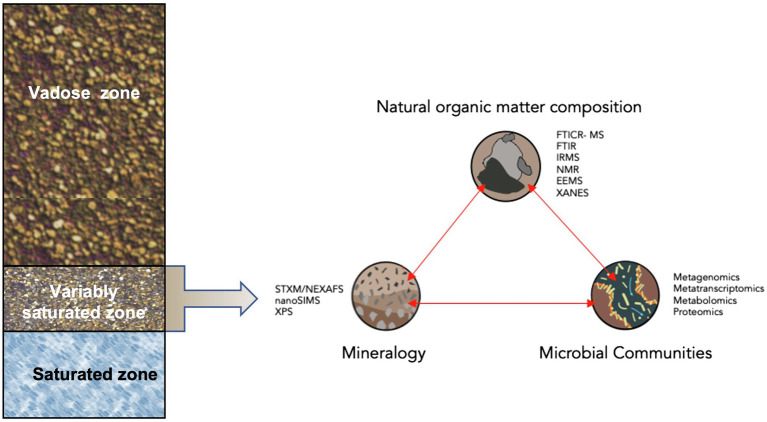
Conceptual diagram of analytical techniques to examine microbial transformations of natural organic matter within the context of organa-mineral interactions in the capillary fringe of the terrestrial subsurface. Analytical techniques include: Scanning Transmission X-ray Microscopy (STXM) with Near Edge X-ray Absorption Fine Structure spectroscopy (NEXAFS), high-resolution secondary ion mass spectroscopy (NanoSIMS), X-ray Photoelectronic Spectroscopy (XPS), Fourier transform Ion Cyclotron Resonance Mass Spectrometry (FTICR-MS), FT Infrared Spectroscopy (FTIR), Isotope Ratio Mass Spectrometry (IRMS), Nuclear Magnetic Resonance (NMR), Excitation-emission Matrices (EEMs), and X-ray Absorption Near Edge Structure Spectroscopy (XANES).

Subsurface environments, roughly 2–10 m below ground surface, can account for more than half of the total soil carbon (Rumpel and Kögel-Knabner, 2011; Kalks et al., 2021). Yet, the transformation of natural organic matter, which is a primary source of C and N for microbial communities, remains poorly understood (Smith et al., 2018b). A particularly crucial zone in the terrestrial subsurface is the capillary fringe, which is a terrestrial–aqueous interface where microbial activities are governed by complex physicochemical interactions due to the solid/aqueous/gaseous interfaces and variable redox conditions that impact microbial community composition on mineral surfaces and groundwater (Figure 1; Zhang and Furman, 2021). Isotopic studies indicate that microbial communities, along with the strength of mineral associations, significantly regulate carbon stabilization (Schrumpf et al., 2013; Mathieu et al., 2015), and surveys of subsurface microbial communities reveal a plethora of key players from under-characterized microbial taxa (Hug et al., 2015; Wu et al., 2020). Furthermore, evidence that species present in low abundances (<0.1% relative abundance) may have disproportionately large impacts on carbon transformation in subsurface environments highlight the vast metabolic unknowns we face in understanding carbon transformations in this subsurface zone (Long et al., 2016). Altogether, understanding the intricate and interrelated microbial and mineral processes that drive NOM formation and stabilization remains a “black box” that requires the application of interdisciplinary approaches, particularly in physiochemically-complex environments like the capillary fringe. With carbon stocks of this magnitude, it is vitally important to consider microbial impacts on their stability and subsequent feedbacks to atmospheric CO_2_ concentrations.

A holistic, systems biology approach, defined here as coupling NOM chemistry with microbial community omics, provides an important avenue to connect microbial pathways to carbon transformations and environmental conditions. Integration and synthesis of all the necessary analytical approaches requires expertise in many different fields, including physical chemistry, organic chemistry, microbial physiology, and bioinformatics, among others. Fortunately, the increasing application of state-of-the-art instruments to probe NOM chemistry, related edaphic factors, and microbial genomic and metabolic data provide opportunities to examine the role of microbial community transformations of NOM on terrestrial subsurface carbon dynamics. However, there are still missed opportunities in the employment of these analytical techniques to collect data in a continuous or non-destructive manner (Figure 1).

Here, we review several state-of-the-art analytical tools used to explore the relationship between microbial community composition and function and NOM transformations by highlighting key technologies and frameworks related to NOM in both dissolved and mineral-bound forms. We then discuss the necessary development of novel tools for performing non-destructive experiments that capture microbial impacts on carbon cycling in the capillary fringe of the terrestrial subsurface and other terrestrial/aqueous interfaces.

## Current Analytical Chemistry Approaches

Establishing mechanistic links between microbial community function and NOM transformation requires a sound understanding of NOM structure and composition. NOM exists as a heterogenous mix of compounds with a wide variety of sizes, chemical compositions, structures, functional groups, and polyelectrolytic characteristics that govern unique reactivities (Baldock and Skjemstad, 2000; Chen et al., 2002; Cooper et al., 2020). These reactivities regulate NOM-mineral associations as well as NOM responses to seasonally dynamic redox environments within the capillary fringe. Dramatic fluctuations of redox potential in the capillary fridge due to the variable water table and the disruptive extraction of sediment from the terrestrial subsurface remain significant challenges in understanding microbial NOM transformations. Specifically, redox conditions affect structure and activity of microbial populations (Blodau and Moore, 2003; Hansel et al., 2008), speciation and bioavailability of metals and nutrients (De Jonge et al., 2012), and sorption and bioavailability of NOM compounds (Marschner and Kalbitz, 2003). Thus, redox conditions can significantly alter the types of interactions that occur in the capillary fringe that impact NOM transformation. However, NOM extraction methods and solvents for downstream analysis, if not chosen carefully, can also introduce uncertainties in interpretation.

For soil samples, NOM must be separated from the mineral phase using solvents and extraction methods that vary in their effective isolation of defined mineral-NOM fractions (von Lützow et al., 2007). NOM characterization of liquid (e.g., groundwater, sediment extracts) and solid samples differs widely. Common extractants to release NOM from mineral surfaces include water (Kleber et al., 2015), sodium pyrophosphate (Ross et al., 1985; Dahlgren, 2015), acid ammonium oxalate, hydroxylamine and sodium dithionite (McKeague and Day, 1966; Wagai and Mayer, 2007).

General patterns on the bioavailability and age of the compounds from these extractions can be made, though estimates may vary depending on the environment from which the samples were collected from. In soil, SOM from organo-metal complexes has been measured to be younger than organic matter extracted from more crystalline mineral phases (Heckman et al., 2018). Furthermore, NOM that is occluded by co-precipitation or adsorbed by pedogenic oxides may be less available to microbes under oxic conditions (Kögel-Knaber et al., 2008). Associations of organic matter on pedogenic oxides, especially more crystalline forms may be less bioavailable given radiocarbon age measurements above. But these stable organic matter interactions could be interrupted. For example, under anoxic conditions, organic matter could be released with the reduction of iron from iron (oxygen)hydroxides, especially less crystalline forms, and with lower C to Fe ratios (Han et al., 2019). Further work needs to be done to determine which fractions of these co-precipitated and adsorbed organic matter pedogenic oxides are more readily released and bioavailable under changing redox conditions. Thus, a significant portion of NOM extracted from reducing solutions could actually be bioavailable in environments with frequent redox changes and cannot be assumed to be protected against microbial degradation. In the capillary fringe zone, investigating these redox-based co-precipitation and adsorption dynamics may be extremely helpful in determining which fractions of NOM from deep soils and sediments may be more bioavailable to microbes. In the following sections, we highlight several analytical methods that have been recently employed in the characterization of terrestrial sediment NOM (Table 1).

**Table 1:** Brief summary of pros and cons for current analytical techniques applied to natural organic matter characterization (Gerin et al., 2003; Holbrook et al., 2006; Godin and McCullagh, 2011; Remusat et al., 2012; Chen and Yu, 2021).

	Pros	Cons
Fourier transform ion cyclotron-mass spectrometry (FTICR-MS)	Detects hundreds to thousands of compounds Enables classification into compound classes (sugar, protein, lipid, carbohydrates)	Qualitative and semi-quantitative Identifying exact formula for large molecules remains difficult
Isotope ratio mass spectrometry (IRMS)	Can be paired with stable isotopes to understand carbon turnover Available for both gaseous and liquid samples	In liquid samples, only inorganic mobile phases can be used limiting the types of compounds that can be analyzed In gaseous samples, derivatization can change isotopic ratios for polar compounds
Nuclear magnetic resonance (NMR)	Quantitative characterization of compounds Non-destructive and applicable to solid, gel, gaseous or solution-state samples Qualitative functional group composition for complex mixtures Multiple atoms that can be analyzed relevant to NOM with appropriate probe (^1^H,^13^C, ^15^N, ^31^P)	Limited in ^13^C and ^15^N by low concentration of these isotopes in soils, sediments, and extracts Historically insensitive technique
Excitation-emission matrices (EEMs)	Useful for analyzing the chemical characteristics of NOM Minimal sample preparation required Uses small sample volumes (0.2–1 ml)	Excitation and emission spectral correction influence fluorescence Qualitative Requires a comprehensive spectral library of reference compounds and semi-quantitative
Fourier transform infrared spectroscopy (FTIR)	Analyzes chemical bonding, molecular environment, and molecular structure of NOM	Direct interpretation is challenging because of high polyfunctionality and complexity of NOM
X-ray absorption near edge structure spectroscopy (XANES)	Element-specific technique that provides electronic and structural information about an element in a compound (e.g., C or N) Minimal sample preparation required Requires low elemental concentrations	XANES regions of complex heterogeneous matrices are difficult to interpret Requires comprehensive spectral library of reference compounds
Scanning Transmission X-ray Microscopy/Near Edge X-ray Absorption Fine Structure Spectroscopy (NEXAFS) with high-resolution secondary ion mass spectroscopy (STXM/NEXAFS with NanoSIMS)	Provides information on microbial binding and spatial distribution on OM and minerals Correlated information on element speciation together with isotopic enrichment level Provides quantitative, *in situ* information on molecular class of mineral-associated NOM Minimal sample preparation Can use lower elemental concentrations	Samples must be mounted in the same fashion for STXM and NanoSIMS analysis Several regions of interest need to be investigated to derive a statistically significant interpretation
X-ray photoelectronic spectroscopy (XPS)	Provides elemental abundance that can cover a range of elements (C, N, Al, Si) Enables adsorption or surface enrichment analysis of soils, clays or minerals	Not ideal for specific spatial distribution Highly sensitive so caution is required to prevent contamination

### Fourier Transform Ion Cyclotron Resonance—Mass Spectrometry

Fourier transform ion cyclotron mass spectrometry (FTICR-MS) is a type of ultra-high-resolution mass spectrometry that can detect and assess the elemental formulas of hundreds to thousands of compounds in NOM (Kujawinski and Behn, 2006; Zhang et al., 2021). To employ this method, NOM must be extracted from soils using carefully selected solvents and extraction methods, as described above. Samples are then ionized *via* electrospray ionization or in combination with other complementary ionization techniques (Cao et al., 2015; Zhang et al., 2021). Detection plates measure (m/z) of the ions based on the ionic cyclotron frequency in a magnetic field (Zhang et al., 2021), and the detected compounds can be classified using a van Krevelen diagram into biochemical compound classes (e.g., lignin, amino sugar, protein, lipid, carbohydrates, tannin, and condensed aromatics) based on their appearance in distinct locations of the diagram (Sleighter and Hatcher, 2007; Minor et al., 2014; D’Andrilli et al., 2015). The versatility of FTICR-MS in studying uncharacterized NOM in soil is useful for connecting NOM composition, particularly microbially-derived components, with microbial communities.

FTICR-MS has been successfully applied to NOM characterization in several terrestrial environments. FTICR-MS metabolite profiling has been used to identify origins of soil NOM, particularly as a result of bacterial transformations (Lechtenfeld et al., 2015; Tfaily et al., 2015), and was instrumental in determining the abundance of microbially-derived products in soil organic matter (Simpson et al., 2007). Additionally, FTICR-MS has been used to demonstrate that higher proportions of phosphorus corresponded to higher rates of biodegradability (de Oliveira et al., 2015), and that oxidized lower molecular weight compounds were transformed much faster than those with higher molecular weight (Malik et al., 2016; Zhang et al., 2021). Furthermore, Wu et al. (2018) applied FTICR-MS to characterize the NOM derived from shallow terrestrial sediment, and demonstrated the utilization of carbohydrates and proteins in sediment NOM by indigenous microbial communities in sediment-associated groundwater.

### Isotope Ratio Mass Spectrometry

Isotope ratio mass spectrometers (IRMS) enable the measurement of variations between isotopic ratios, such as ^13^C/^12^C. There are several methods to direct samples into the IRMS such as gas chromatographs or elemental analyzers. Elemental analyzers in combination with IRMS (EA-IRMS) produces representative data of an average isotopic signal of the sample (Muccio and Jackson, 2009). Stable C isotope composition (δ^13^C) of NOM, which is measured by elemental analysis – isotope ratio mass spectrometry (EA-IRMS), provides insight into the source, composition, and turnover of organic carbon (OC) in soils and sediment. The δ^13^C of OC in soils has been linked to associated plant δ^13^C signatures. For example, soil 0-30 m predominantly reflects the original isotopic signal of the former vegetation (Vinnepand et al., 2020), and the δ^13^C_(OC)_ of subsurface rhizosphere soil is usually higher than that of surface soil, as the δ^13^C of root material is generally higher than that of the above ground biomass, such as leaves (Wang et al., 2018). However, microorganisms also contribute to δ^13^C_(OC)_ variations in soils. Microbes, invertebrates, and other soil fauna are typically enriched in δ^13^C compared to source-substrates, and their activities and biomass may also affect the turnover and δ^13^C of soil OC (Wang et al., 2018). Thus, the vertical distribution of the δ^13^C from shallow to deep mineral soil (>10 m) can be indicative of microbial preferences for ^12^C vs. ^13^C in decomposing substrates, which in turn increases the ^13^C/^12^C of residual OC fractions (Wang et al., 2018). Therefore, δ^13^C_(OC)_ in soil and sediment tends to increase with depth until it reaches a maximum value at which point it approaches an asymptote.

It is important to note that, although MS techniques provide high resolution data on the diversity of compounds found in NOM, there are some limitations. MS techniques provide mostly qualitative and semi-quantitative information on the composition of NOM. For example, in EA-IRMS, the data represent an average of the isotopic ratio and is not able to differentiate the contribution of each isotope to the total, making it largely a qualitative method (Muccio and Jackson, 2009). The relative abundance of detected ions is not only influenced by the concentration of compounds present, but also by their ionization (Fenn, 1993). These limitations can be overcome by combining MS analysis with more quantitative spectroscopy or spectrometry techniques to gain a more comprehensive picture.

### Nuclear Magnetic Resonance

Nuclear magnetic resonance (NMR) characterizes NOM in soils and sediments quantitatively. This technique can focus on significant atoms within NOM, such as ^1^H, ^13^C, ^15^N, and ^31^P with minimal disruption of the sample. Several studies have utilized NMR to determine NOM composition of solid, gel, or solution-state samples (Simpson et al., 2012). Simpson et al. also showed that NOM in soil samples was comprised of a diverse range of compounds such as proteins, carbohydrates, and lipids. Furthermore, one-dimensional and two-dimensional NMR experiments indicate that NOM found in soils was primarily made up of plant- and microbially-derived compounds along a decomposition spectrum (Simpson et al., 2007; Lam and Simpson, 2008; Miltner et al., 2009), with significant fractions of NOM derived specifically from microbial metabolites and necromass (Kögel-Knabner, 2017). Similarly, Knicker and Lüdemann (1995) used NMR to determine the transformations of organic nitrogen with increasing decomposition by microorganisms, and showed that the majority of organic nitrogen was not heterocyclic condensed N but rather composed of plant and microbial proteins and amino acids.

NMR can also provide information on functional groups for complex mixtures or structural information for single compounds by analyzing multiple atoms in a single sample. For example, initial characterization of NOM composition can focus on ^1^H atoms before using ^13^C or ^15^N to concentrate on specific chemical groups within the same sample. This application is critical for multidimensional experiments, such as Heteronuclear Single Quantum Coherence or Heteronuclear Multiple Quantum Coherence (HSQC/HMQC) that use cross-peaks of two atoms (such as ^1^H connected to either ^13^C or ^15^N) to provide more information of the specific chemical groups in NOM. Larger macro molecules or aggregated NOM can be characterized *via* Diffusion-edited ^1^H NMR (DE) that takes advantage of molecular self-diffusion in a sample solution (Simpson, 2002). These applications can provide information on transformations of NOM as a response to nutrient inputs, disturbance, time, mineral composition, and many other factors to gain insight into the terrestrial C cycle. For example, NMR has been used to quantify a change in NOM chemistry in two tropical forest soils after N-fertilization with corresponding shift in microbial community composition (Cusack et al., 2011). Tracking the outcomes of specific components of NOM, such as of ^13^C- and ^15^N-labeled protein, after interactions with Mn oxide revealed an abiotic pathway in generating small polypeptide fragments that may be available to microorganisms in environments with reactive pedogenic oxides (Reardon et al., 2016). Thus, NMR can be exploited by implementing stable isotope probing (SIP) to detect transformations of ^13^C- or ^15^N-labeled compounds in soil or sediments with greater resolution and less analysis time. However, paramagnetic compounds, such as Fe, can significantly decrease the resolution of NOM analysis. While the addition of hydrofluoric acid can dissolve Fe and other mineral phases in order to increase the concentration of NOM (Schmidt et al., 1997), this approach can also result in loss of carbohydrate signals.

### Excitation-Emission Matrices

Fluorescence spectroscopy, including the generation of excitation-emission matrices (EEMs) has proven useful in characterizing the chemical quality of NOM (Cory and McKnight, 2005; Fellman et al., 2010). Fluorescence characterization of organic matter is based on detecting the optically active fractions of organic matter that differs between compositionally distinct organic matter compounds (Li et al., 2020). Beams of light within the fluorescence spectrometer excite electrons within organic matter compounds in order to emit light, which is measured by a detector. Different analytical methods to interpret EEMs are available to qualify and quantify NOM (Li et al., 2020). Most notable are principal component analysis (PCA) and parallel factor analysis (PARAFRAC), which allow for more detailed qualitative analysis in contrast to older methods like peak picking and fluorescence regional integration (Li et al., 2020).

Several studies have utilized EEMs in groundwater and terrestrial sediment NOM characterization in combination with other physicochemical analyses to draw connections among NOM qualities and environmental conditions (Mladenov et al., 2010; Li et al., 2019; Huang et al., 2021). Huang et al. utilized EEMs to characterize NOM in groundwaters with varying ammonium concentrations, finding that high-ammonium groundwaters exhibited increased humic-like signatures as compared to low-ammonium groundwaters, abundant in protein-like signatures (Li et al., 2019). In addition to nutrients such as ammonium, mobilization of metals is a critical facet of environmental conditions that affect microbial activity in variable redox conditions. Metals and metalloids such as iron and arsenic have been extensively studied in variable redox zones and can be evaluated as examples of how NOM interacts with metals in the capillary fringe. Mladenov et al. (2010) demonstrated that groundwater collected at 11–14 m BGS exhibited lignin-like and aromatic NOM signatures, and that iron and arsenic mobilization may be associated with microbial consumption (and therefore absence) of labile NOM in the subsurface. Li et al. (2019) further studied the effects of NOM on arsenic mobilization in the subsurface, demonstrating that humic-like NOM participates in arsenic complexation, further driving arsenic mobilization forward.

One of the advantages of fluorescence technology is its high sensitivity and selectivity (Li et al., 2020). Fluorescence excitation-emission matrices have grown in popularity because of the large amount of data they generate (visual maps and multidimensional information) and the ability to understand temporal and spatial dynamics of NOM (Fellman et al., 2010; Li et al., 2020). However, fluorescence overlap due to different types of organic matter occurring in similar regions of the spectrum can hinder accurate determination of NOM compounds in the sample. Additionally, peak shift, where similar types of organic matter are identified as having different fluorescence can complicate interpretation of data (Li et al., 2020).

### Fourier Transform Infrared Spectroscopy

Fourier transform infrared spectroscopy (FTIR) is a technique that analyzes the vibrations of molecular bonds at infrared wavelengths to understand the molecular composition of compounds. FTIR can be used to identify organic matter functional groups because each spectrum is the result of unique absorption bands of different molecular bonds (Ellerbrock and Gerke, 2013; Orhan-Yanıkan et al., 2020). Furthermore, FTIR can be used to understand the relative proportion of different OM functional groups, as different regions of the infrared spectra correspond to different compound classes (Orhan-Yanıkan et al., 2020). There are several FTIR sample preparation techniques that offer different advantages and disadvantages depending on experimental needs (Ellerbrock and Gerke, 2013). For example, in the attenuated total reflection (ATR) technique, samples are placed between polished crystal plates of diamond, Germanium or ZnSe and no chemical additions are required. In contrast, the potassium bromide technique requires mixing finely ground samples with potassium bromide, providing highly resolved and easily interpretable spectra at the risk of potentially shifting absorption bands (Ellerbrock and Gerke, 2013).

Fourier transform infrared spectroscopy provides an avenue of exploring how the chemical structure of soil or sediment organic carbon influences carbon stabilization (Fox et al., 2017). FTIR spectrum resolution can be improved using methods that separate NOM from minerals; this also allows for the separation of different pools of NOM that may have varying solubilities and reactivities that impact mineral association and stability. Highly mobile and labile NOM were found to have spectra with large C-H peaks and higher fractions of carboxylic acid groups than metal-stabilized NOM fractions (Fox et al., 2017). Additionally, FTIR can be used to understand how mineral properties interact with NOM chemical structures. Spence and Kelleher examined kaolinite mineral interactions with organic matter to understand what compounds are likely accessible to microbes as opposed to compounds that are likely to become physically protected through strong associations at the mineral surface. Strong absorption on kaolinite surfaces at 3,408–3,300 cm^−1^ indicated the presence of phenolic, carboxylic groups, and amides, suggesting a high degree of stabilization of microbial-derived components that could be instrumental in forming stable mineral-organo complexes (Spence and Kelleher, 2012). These applications of FTIR can often complement many techniques to characterize NOM, such as X-ray-based techniques.

### X-ray Absorption Near Edge Structure Spectroscopy

Currently, synchrotron-based spectroscopic approaches are extensively used to investigate the functional group composition of NOM at the molecular-scale. Bulk X-ray absorption fine structure spectroscopy (XANES) is an element-specific technique that yields electronic and structural information about the element of interest (e.g., C and N in case of NOM). Air-dried samples can be analyzed by XANES, and this minimally disruptive sample preparation makes this analytical technique increasingly popular. An average XANES spectrum is produced by calculating the weighted sum of many reproducible spectra containing intense resonance features representing chemical attributes (e.g., functional group composition, oxidation state) of all compounds in a sample at a specific energy edge (Fendorf and Sparks, 1996). There are two basic methods used to estimate NOM composition in an unknown sample from a XANES spectrum. The “fingerprint” approach directly compares the sample spectrum with spectra of known reference compounds, enabling the assignment of spectral features characteristic of the respective C or N-containing moieties (e.g., carboxyls, amides, aromatics, and phenols). Comprehensive libraries of C and N XANES spectra of environmentally relevant pure reference compounds containing C, N, or both are available in the literature (Leinweber et al., 2007, 2010). However, in complex heterogeneous matrices such as soils or sediments, the XANES regions of different species often overlap significantly over the entire spectral region, requiring a careful deconvolution of the spectra using known reference compounds. Whereas feature assignment by comparison with reference spectra yields qualitative information, quantitative information on sample composition can be achieved by spectral deconvolution into sub-peaks using Gaussian or additively linked Gaussian–Lorentzian functions (Ravel and Newville, 2005). Additionally, low NOM concentrations in deeper subsurface sediments often pose a challenge for good spectral quality and hence several XANES scans need to be collected for each sample. For such samples, conducting XANES on NOM fractions isolated from bulk sediments yields a better signal. Finally, C XANES features indicative of metal-NOM complexation (typically occurs at ~287.5 eV for polyvalent metal complexation with C) also provides information on the potential organo-mineral associations in subsurface sediments. The following section discusses an even more advanced approach of using highly resolved spectromicroscopy technique to elucidate NOM composition and mineral-organic interactions in deeper sediment samples.

### Scanning Transmission X-ray Microscopy With Near Edge X-ray Absorption Fine Structure Spectroscopy in Combination With High-Resolution Secondary Ion Mass Spectroscopy

Stable isotope tracer experiments followed by NOM and mineral imaging with high-resolution secondary ion mass spectroscopy (NanoSIMS) can be an effective way to address critical characteristics of soil microscale processes, such as the source of mineral associated NOM, relative importance of biotic and abiotic processes in NOM stabilization in soils, and spatial variance in these processes (Pett-Ridge and Weber, 2012; Keiluweit et al., 2015). NanoSIMS is based on generating a beam of primary ions (Cs^+^ or O^−^) to excite the sample surface in order to produce secondary ions (Mueller et al., 2013). These secondary ions are positive or negative depending on the charge of the primary ion used [i.e., negative secondary ions are generated when using (Cs^+^)]. NanoSIMS imaging, can detect secondary ions to a lateral resolution of up to 50 nm (Mueller et al., 2013). Although it is a highly complex approach, NanoSIMS been used with increasing regularity in terrestrial systems, and has provided evidence for the accumulation of organic rich compounds of microbial origin binding directly to minerals, spatial distribution of OM binding within aggregates (Pett-Ridge and Weber, 2012; Solomon et al., 2012; Keiluweit et al., 2015; Eusterhues et al., 2020; Seyfferth et al., 2020).

Far more uncommon is the application of NanoSIMS in concert with Scanning Transmission X-ray Microscopy (STXM) coupled with Near Edge X-ray Absorption Fine Structure spectroscopy (NEXAFS; Remusat et al., 2012). In STXM, monochromatic X-rays pass through a focusing lens to a section of a sample. X-ray absorption is measured based on the sample position and the detection of transmitted X-rays (Stuckey et al., 2017). STXM is often used in combination with NEXAFS, which is a type of absorption spectroscopy. In NEXAFS, an X-ray photon is absorbed by a core level of an atom in the sample resulting in the emission of a photoelectron. The hole left by emission can be filled using an electron from another shell, which makes NEXAFS ideal for understanding the bonding environment of atoms (Vlasov et al., 2012). The ‘STXM-SIMS’ analytical approach has the unique capacity to yield quantitative, *in situ* information on the molecular class and elemental quantity of mineral-associated NOM. When combined with isotope labeling, STXM/NEXAFS and NanoSIMS imaging can be used to infer the molecular and spatial fate of the added labeled material in a mineral matrix, and can contribute to a mechanistic understanding of sorption, occlusion, and decomposition processes that operate at fine spatial scales in natural environments. Applied in sequence to the same regions of interest, NanoSIMS is used to determine the spatial fate of the isotopic label while STXM/NEXAFS is used to determine the chemical transformation of the label. Isotopic data are expressed in atom percent excess (APE), with higher APE representing higher isotopic enrichment. Similar to bulk XANES, linear combination fitting of the average C and N NEXAFS spectra using reference helps to identify the molecular composition of NOM while simultaneously providing its spatial distribution with other NOM moieties. This technique has been successfully used to look at microbial transformations of organic residues from root exudates in natural samples (Keiluweit et al., 2015). Overall, the STXM-SIMS approach helps to identify the fate of the isotopically labeled substrate as it gets bound to minerals or undergoes microbial metabolism thereby leading to differential NOM degradation pathways in deeper subsurface environment depending on the redox status.

### X-ray Photoelectronic Spectroscopy

The final tool to investigate surface chemistry of soil and sediment particles we will highlight here is X-ray photoelectronic spectroscopy. Within a vacuum, samples are mounted on holders and particles from soils or sediments can be irradiated with X-rays, which causes photoelectron emissions of the atoms on the top 2–10 nm of the surfaces (Amelung et al., 2002; Woche et al., 2017). This allows researchers to specifically observe NOM attached on sediment or soil minerals. XPS photoelectron emission can provide elemental abundance with a wide scan of 1–1,200 binding energy that can cover N, C, Al, Si, and other elements. When focusing on the binding energy of a specific element, XPS can provide functional group information of core electrons on the surface of soil or sediment particles (Arnarson and Keil, 2001; Amelung et al., 2002; Mikutta et al., 2009). The use of XPS and other techniques have revealed that the OM in marine sediments is clumped in spots on the surface and that OM binds sediment particles together into aggregates (Arnarson and Keil, 2001). Similar results have been observed in soils as well, and specific compounds in contact with mineral surfaces have been reported as enriched with aromatic carbon with outer regions enriched with amines/peptides (Amelung et al., 2002; Mikutta et al., 2009). The use of XPS in combination with multiple techniques can elucidate spatial and temporal dynamics of NOM on soils and sediments. However, analysis regions are limited to small portions of the sample, requiring multiple scans to ensure C and N spectra are representative of the entire sample surface.

## Integrating Multi-omics Analyses for Pathway Characterization

Subsurface environments contain over half of all global microorganisms, yet less than 8% of available 16S rRNA gene sequences have been derived from subsurface organisms and even fewer are represented by complete genomes, severely limiting our capacity to understand the interconnected microbial metabolic processes that drive carbon transformations (Anantharaman et al., 2016; Long et al., 2016; Lohmann et al., 2020). Therefore, it is critical to continue characterizing subsurface environments using not only genomic analyses, but a suite of omics tools. Microbial capacity for carbon transformations is heavily impacted by the temporal and seasonal dynamics (i.e., in environments such as the capillary fringe) that alter microbial diversity (Long et al., 2016; Smith et al., 2018b), especially in the distribution of planktonic and biofilm microbial communities in aqueous pore spaces and attached to mineral surfaces, respectively. Biofilm-dwelling microbial communities are considered to dominate subsurface environments due to the advantage biofilm matrices provide in terms of water and nutrient retention, metabolic cooperation, and protection from rapid fluctuations in the environment (Smith et al., 2018b). Often, studies attribute microbial transformations to the “subsurface microbial community,” despite differences between planktonic or biofilm microbial communities in carbon utilization (Hazen et al., 1991), nutrient acquisition (Ahmed and Holmström, 2015), and functional capacity (Griebler et al., 2002; Lehman, 2007; Smith et al., 2018b). Few studies have systematically compared these two populations (Lehman et al., 2001; Smith et al., 2018b), yet attributing potentially unique carbon transformations between planktonic and biofilm microbial is essential for developing an accurate representation of how these distinct microbial populations impact subsurface NOM transformations.

The connections between microbial community function and the dynamic fluctuations in capillary fringe water content, oxygen availability, and substrates are difficult to identify without merging analytical chemistry and an omics-level perspective (Smith et al., 2018b). These integrated, systems-level insights are critical to examining the underlying metabolic links that drive carbon cycling and foundational to developing process-based models of biogeochemistry in dynamic areas of the subsurface. Recent studies have increasingly incorporated NOM analytical chemistry and microbial omics to form a more cohesive understanding of the interactions between NOM structural complexity, mineral surfaces, and microbial function (Cooper et al., 2020). The following section illustrates the utility of applying approaches to characterize NOM with microbial data at various levels (genomic, transcriptomic, proteomic, metabolomic) to facilitate the connection of NOM transformation to microbial community structure and function in the terrestrial subsurface.

### Pre-translational-Omics

Metagenomics provides an opportunity to understand the functional potential of difficult-to-cultivate microorganisms and can illuminate potential interactions among microbial community members that drive biogeochemical cycles of carbon in subsurface environments (Anantharaman et al., 2016; Long et al., 2016). Genome-resolved metagenomics is critical for determining potential metabolic interactions among difficult-to-observe microorganisms and forms the basis of interpreting metatranscriptomic and metaproteomic data (Long et al., 2016). Metagenomic studies have verified the syntrophic nature of subsurface microbial communities (Anantharaman et al., 2016), the importance of less abundant species in carbon transformations (Long et al., 2016), and the truly wide multitude of unknown and rare species that have only been observed thus far using metagenomic approaches (Anantharaman et al., 2016; Hug et al., 2016; Long et al., 2016; Probst et al., 2018; Smith et al., 2018b). Several studies have demonstrated the utility of coupling metagenomics with NOM characterization techniques discussed previously.

In a multi-omics study that also employed FTICR-MS to determine chemical diversity of sediment NOM, Graham et al. (2018) observed varying phenotypic plasticity in microbial communities depending on the types of metabolites available. FTICR-MS detected strong spatial differences in protein- and amino sugar-like compounds between “hotspots” in the sediment where there were highly diverse organic matter compounds and “low-activity” sediments where diversity was lower. Metagenomics of both hotspot and low-activity sediments indicated similar microbial composition, but metabolomic data indicated higher instances of inorganic N cycling in hotspot areas that inadvertently also released labile C (Graham et al., 2018). Similarly, a three-year study of NOM paired with microbial community analysis indicated that shifts in NOM composition and shifts in bacterial community composition were correlated (Benk et al., 2019). Ultrahigh resolution MS indicated that dissolved organic matter (DOM) in groundwater was in order of higher proportion composed of: highly unsaturated molecules, unsaturated aliphatics, polyphenols, and peptide-like compounds. In combination with carbon dating, Benk et al. found that the amount of highly unsaturated molecules decreased by about 10%, with a corresponding increase in aliphatic and peptide-like substance. This signature is indicative of a shift from plant-derived DOM to microbial-derived DOM, solidifying the idea of continuous microbial transform of DOM over time (Benk et al., 2019). Through merging an understanding of the carbon sources available with the corresponding microbial community, we can better understand how these carbon sources are transformed in response to microbial community activity and examine the role of microbially-derived organic matter on subsurface carbon cycling.

Transcriptomics can be used to examine the pattern of gene expression during monitored physiological activities and in response to environmental conditions (Konopka and Wilkins, 2012; Gushgari-Doyle and Alvarez-Cohen, 2020). There are very few studies, in any environment, that have coupled transcriptomics with complex organic matter characterization to identify correlations among the datasets. McCarren et al. (2010) utilized a marine system to evaluate the effects of amending high molecular weight DOM on community gene expression. McCarren et al., showed that addition of DOM stimulated expression of different genes related to phosphate and nitrogen assimilation, chemotaxis, and motility from different taxa, especially *Idiomarina* and *Alteromonas* spp., relative to an unamended control. These results indicate taxa-specific resource partitioning of carbon sources that have downstream consequences for succession, metabolic pathways, and the carbon cycle. One application of this type of analysis in the subsurface is to examine relationships between specific organic matter compounds in various redox conditions to identify consequences to microbial community transcription. Such a study would provide a glimpse of how microbial community activity changes in response to redox-related impacts on carbon resources. In fungal microbiology, Nicolás et al. (2019) utilized FTIR-MS and XANES in combination with transcriptomics to characterize soil organic nitrogen mobilization by ectomycorrhizal fungi isolates, finding that carbon availability regulated nitrogen mineralization and that patterns of gene expression related to decomposition were unique between the two species examine. In subsurface environment, such techniques could be used to explore gene expression patterns in different taxa in response to organic matter resources as a way of indicating potential microbial niches. However, there are several limitations of transcriptomics that may influence the general absence of studies. First, transcriptomic analyses are highly sensitive to temporal fluctuations due to the short lifespan of RNA (Blazewicz et al., 2013; Arsène-Ploetze et al., 2015). Additionally, transcripts can be difficult to map back to specific members in complex microbial communities. Thus, the utilization of transcriptomics in environmental enrichments or field studies may be difficult to interpret. It is for reasons such as these that researchers may have been inclined to utilize post-translational -omics techniques in lieu of transcriptomics to draw connections among microbial activities and NOM transformations.

### Post-translational-Omics

Microbial (meta)proteomics can be used to characterize all proteins expressed at a given time point to understand how microbial isolates and communities respond physiologically to the environment (Wang et al., 2016). Proteins reflect active translation, and the high accuracy of proteomic approaches enables tracing metabolic processes to specific microbial strains, popularizing the use of metaproteomics to examining microbial roles in nutrient cycling and organic matter degradation (Wang et al., 2016). Proteomics has been widely used in isolate studies to elucidate mechanisms due to the simplicity of axenic culture. For example, Wilkins et al. utilized proteomics in combination with 2D-LC-MS/MS to identify temporal shifts in carbon utilization patterns from early, middle, and late-stage acetate transformation. Early stages were characterized by high abundance of proteins related to biosynthesis as *Geobacter* cells proliferated while middle stages, were characterized by an increased carbon flux through respiratory pathways and high abundance of related proteins for energy generation (Wilkins et al., 2013). Here, Wilkins et al. demonstrated the correlation between microbial protein expression and external, geochemical conditions, particular shifts in carbon resources. However, like transcriptomics, proteomic analyses have been more commonly used in conjunction with NOM characterization chemistry in non-terrestrial matrices, especially marine systems. Han et al. utilized a suite of -omics techniques, including proteomics, with FTICR-MS to observe shifts in the core proteome of a coastal community in response to changes in DOM fractionation due to phytoplankton blooms (Han et al., 2021). Although there were similarities in core proteome expression, metabolic data indicated that available carbon resources (amino acids, lipids, and nucleotides) were preferentially taken up by *Polaribacter marinivivus* and *Lentibacter algarum* while other metabolites were utilized more readily by *Litoricola marina*. These results illustrate both metabolic niche partitioning in response to carbon resources and also the adaptive rapid expression of nitrogen uptake proteins in response to algal blooms of these bacteria.

Proteomic stable isotope probing has also been utilized in characterizing complex OM, particularly in marine environments. Proteomic SIP relies on using a heavy isotope-labeled substrate (i.e., ^13^C or ^15^N) to track incorporation into different biomolecules (Taubert et al., 2012), providing insight into which microbial populations are utilizing a particular substrate and the resulting metabolic byproducts. In marine environments, a large proportion of DOM originates from phytoplankton and is assimilated through microbial transformations (Kieft et al., 2021). Proteomics, in combination with ^13^C-labeled complex DOM, was used to determine how various marine microbial populations assimilated different types of DOM (diatom lysate, diatom exudate, Cyanobacteria lysate, Cyanobacteria exudate) in a series of microcosm studies. Kieft et al. found that although taxonomy remained the same throughout the microcosms, there were strong differences in protein labeling that were indicative of differentiated carbon use capabilities. *Bacteroidetes* preferentially increased ribosomal protein expression in response to DOM from diatom lysates, and many newly synthesized proteins in response to diatom lysates were associated with degradation of carbon-rich high molecular weight DOM (Kieft et al., 2021). In contrast, *Alphaprotebacteria*, highly expressed transport proteins known to target polyamines, nucleosides, amino acids, and other small molecules characteristic of phytoplankton exudates and cyanobacterial exudates, suggesting that this class specializes in a particular DOM faction (Kieft et al., 2021). One application of this dataset was the development of class-specific rates of carbon assimilation to potentially predict the consequences of resource preferences for carbon assimilation or respiration during phytoplankton blooms (Kieft et al., 2021). Studies like these demonstrate the utility of proteomics to examine shifts in carbon flux as a function of microbial physiology, and could yield results critical for understanding carbon transformations in subsurface environments.

Metabolomics, the final member of the -omics analysis suite, involves the comprehensive profiling of the compendium of small molecules in a biological sample (Bowen and Northen, 2010). Metabolomics can provide insight into the actions of translated proteins and additional physiological information to connect the activities of microorganisms to NOM transformations. Smith et al. (2018a) demonstrated the use of exometabolomics with fluorescence spectroscopy (*via* EEMS) and various mass spectrometry techniques (including FTICR-MS) to investigate the biological transformation of freshwater NOM by a *Janthinobacterium* isolate. Similarly, EEMS was applied to analyze the composition of dissolved organic carbon (DOC) and the impact of various recharge waters on microbial respiration in a shallow aquifer. Cooper et al. (2016) found that there were variations in DOC concentration and composition between rainfall, throughfall, stem flow, overland flow, and dissolution holes that bring in water to the vadose zone. Variation in DOC affected the amounts of bioavailable compounds for microbial respiration, and was independent of whether DOC was of terrestrial or microbial origin. Ultimately, the degree to which microbial respiration increased in response to various sources of water was highly dependent on the DOC composition and concentration (Cooper et al., 2016).

The development of omics tools enabled unprecedented abilities to unravel microbial community responses and feedbacks to environmental conditions. Metagenomics is ideal for capturing the genomic capabilities of microbial communities (Anantharaman et al., 2016; Long et al., 2016), particularly if used in conjunction with cultivation-approaches that test expected genotype–phenotype-functional relationships (Wu et al., 2020). Transcriptomics, although not as commonly used in the subsurface, can yield critical insights into active gene expression in response to environmental conditions (Konopka and Wilkins, 2012; Gushgari-Doyle and Alvarez-Cohen, 2020). Proteomics and metabolomics are considered more direct indicators of microbial activity because they are the actively expressed proteins and metabolites, respectively. Proteins can provide a glimpse of metabolic pathways while metabolomics can be used to identify the change in metabolites present as a result of microbial community activity (Bowen and Northen, 2010; Wang et al., 2016). More common recently is the integration of these into multi-omics analysis in conjunction with NOM characterization. These approaches have the potential to yield incredible insights in the transformation of NOM in the redox-sensitive capillary fringe. Advances in analytical techniques open many avenues for microbial community and NOM characterization. Increasing efforts to resolve functional differences between planktonic and biofilm microbial communities enable a more nuanced understanding of which members of different microbial communities impact various aspects of mineral-OM interactions in the capillary fringe (Griebler et al., 2002; Ahmed and Holmström, 2015; Smith et al., 2018b). In the next section, we review new developments in nondestructive tools to decipher microbial impacts on complex carbon transformation are underway that may prove promising in providing *in situ* data.

## Development and Implementation of Novel Tools

While the current approaches that integrate analytical chemistry with omics tools have advanced our understanding of microbial NOM transformation in many environments, one primary obstacle with most of these analytical approaches as applied to the terrestrial subsurface is that they are destructive, e.g., samples must be extracted or experimental replicates sacrificed to obtain data for a specific timepoint during the course of an experiment. The further development of non-destructive and continuous, *in-situ* experimental approaches is paramount for linking microbial activity to terrestrial biogeochemistry.

Non-destructive approaches employing compound-specific labels (e.g., ^13^C/^15^N-labeled organic compounds) coupled to trace-gas analysis have a long history of use in soil biogeochemistry and microbiology (Stevens et al., 1993; Staddon, 2004). The use of natural-abundance isotope, isotopologue, and isotopomer measurements on gaseous fluxes of CO_2_, CH_4_, or N_2_O also provide detailed analysis of potential microbial pathways leading to the transformation and release of those gases (Wankel et al., 2017; Joseph et al., 2019). However, coupling soil processes, as informed by trace gas measurements (e.g., respiration, decomposition, denitrification), with the metabolisms underpinning the process remains reliant on destructive approaches. The collection and measurement of microbially-derived volatile organic compounds (mVCs) shows promise as a non-destructive, surrogate measurement of *in situ* microbial metabolism (Netzker et al., 2020). These mVCs are produced by a broad range of biosynthetic pathways (primary metabolism, fermentation, fatty acid biosynthesis) which gives rise to high structural diversity (Schulz et al., 2020; Weisskopf et al., 2021). Identification of different mVCs can be made through comparison to existing databases of microbial primary and secondary metabolisms (e.g., KEGG). Proton transfer reaction-time of flight-mass spectrometry was recently used to measure microbial volatiles and show that snowmelt increased anaerobic degradation of quaternary amine osmo/cryoprotectants. This study illustrated the value of using volatile emissions as indicators of microbial metabolism (Kim et al., 2021). However, benchmarking mVC detection requires comparison to a range of measurements, including trace gas fluxes, and, for the time being, metagenomic and metatranscriptomic measurements. Even so, mVCs show particular promise for integrating microbial metabolisms within variably saturated regions, below the vadose zone. This region has low to no root density, and as such plant-derived VOCs would not interfere with the microbial signal. Furthermore, predictable changes in water table height driving strong redox dynamics (predictable through seasonally recurrence or *via* the installation of sensors) will prompt clear changes in the activity of different pathways, thus strengthening the signal:noise ratio. In addition to redox dynamics, mVCs can also vary based on environmental parameters including temperature, humidity, and pH (Weisskopf et al., 2021), making this parameter even more useful for studying microbial metabolisms in the capillary fringe.

The application of nondestructive methods to observe microbial dynamics offers many opportunities determine the drivers of microbial transformation of organic carbon. However, accounting for the many edaphic factors that can influence microbial metabolisms remains a difficult task at the field scale. Lab scale microcosms offer an opportunity to focus on specific aspects in order to derive field-relevant insights of the system as a whole. At the lab scale, the development of three-dimensional, nanofabricated landscapes offers an avenue to mimic soil, and recreate the enormous spatial-temporal heterogeneity measured in these environments within microfluidic devices (Aleklett et al., 2018). These devices can represent the response of both spatially-separated, and interacting populations to better address how they respond to fluctuations in the local environment, brought about by pulsed perturbations (Mathis and Ackermann, 2016), resource gradients (Keymer et al., 2006), or nutrient-rich hot-spots (Stocker et al., 2008; Nguyen et al., 2021). For example, Borer et al. (2018) employed a porous microfluidic device to examine spatial organization of intermixed bacterial species across gradients in oxygen and carbon that can form within soil aggregates. The bacterial species included a fluorescently tagged obligate aerobe, and a facultative anaerobe. This study demonstrated that antithetical gradients in carbon and oxygen impose spatial structure on microbial communities with implications for carbon cycling and greenhouse gas production (Borer et al., 2018). In another study the authors imposed a spatially-resolved nutrient gradient on a single interacting *Escherichia coli* population (Keymer et al., 2006). Cells were inoculated into a microfluidic device containing multiple interconnected habitat patches representing a gradient from high-nutrient/low stress environments to low nutrient/high stress ecosystems. The authors observed the formation of a metapopulation, interacting subpopulations exhibiting dynamic spatial distribution, which reflects rapid acclimation to more stressful conditions to avoid competitive displacement from to nutrient rich habitats.

Microfluidics show clear promise as a technology to study the rules regulating spatial distribution across gradients or under perturbed conditions. Further development is required to identify the metabolic responses of microorganisms to perturbation and the traits underpinning these responses. As materials used for the construction of microfluidic devices evolve from the polydimethylsiloxane to glass and silicon more sophisticated techniques can be used to profile metabolic changes occurring in populations. For example, the use of quantum NMR (Smits et al., 2021) or synchrotron-based, Fourier-Transform infrared (FTIR) microspectroscopy (Holman et al., 2010), both show promise for characterizing microbial metabolism across spatio-temporal scales. Synchrotron-based FTIR is an ultra-sensitive, non-invasive approach to measure changes in the cellular chemical environment within cells in real-time. At the most basic level, all bacterial systems are composed of water, lipids, proteins, and carbohydrates. This SR-FTIR approach has been used previously to characterize changes in this biochemical composition within fluctuating environments (Holman et al., 2009). Combining such an approach with the spatial gradients of microfluidics provides high-resolution information on how microbial cells distribute across gradients, and how perturbations (such as rapid changes in soil moisture attributable to a fluctuating water table) shapes microbial metabolism, and hence necromass.

## Discussion

Critical advancements in analytical chemistry have brought the study of NOM transformation to the forefront of environmental research. In addition, development of omics techniques to probe microbial community activity has shed light on microbial impacts on soil biogeochemistry. Yet, applying these analytical techniques and methods at appropriate scales to capture microbial impacts on ecosystem processes remains a major challenge. The stratified nature of the subsurface and porous nature of the soil environment result in a highly heterogenous environment characterized by pore spaces with variable saturation, oxygen, and chemical properties (Brockman et al., 1997). Furthermore, mixing of water and gas throughout the sediment creates strong redox gradients that alter physical and chemical properties from distances of centimeters to meters (Brockman et al., 1997). These processes directly impact microbial communities through changing redox conditions, moisture availability, and nutrient flux (Smith et al., 2018a). Thus, micro-scale heterogeneity ultimately impacts microbial activity, including the capacity to transform organic matter. Studies at centimeter scales indicate that microbial distribution can be highly variable in subsurface sediments over centimeter distances (Brockman et al., 1997), and that microbial diversity changes with spatio-temporal variation of groundwater and sediment chemistry (Smith et al., 2018a; Khurana et al., 2022). However, methods to measure pore scale processes and scale those observations to field-relevant are still under development. Microfluidics, which enables full control of fluid flow can be paired with natural substrates or configured to mimic natural microenvironmental conditions (Jimenez-Martinez, 2022). Thus, it can provide a means to observe how physical and chemical heterogeneity impacts microbial populations (Vos et al., 2013; Jimenez-Martinez, 2022). Furthermore, micro-computed tomography allows visualization of the soil structure at micrometer resolutions, which can enable studies that examine the impact of different aggregate structures on microbial communities (Vos et al., 2013). Lastly, NanoSIMS (described above) can be used to determine the distribution of organic matter in aggregates and microbial cells (Vos et al., 2013). Applying the analytical techniques we describe here to scale-relevant samples when feasible or microcosm-based designs that explicitly test spatial effects on microbial communities could help shed light on patterns that are amenable to scaling up to field-relevant scales.

We propose that employing analytical chemical techniques in conjunction with omics methods can form a systems biology approach to comprehensively understand microbial NOM transformation in the terrestrial subsurface. Although there are benefits and disadvantages of each technique, coupling these analytical chemistry with -omics technologies has proven fruitful in several environmental systems, especially in marine ecosystems, and can be used to make equally beneficial discoveries in NOM biotransformation in the capillary fringe. However, there are several challenges intrinsic to the capillary fringe that must be considered. These include, but are not limited to, variable redox conditions, contributions of planktonic vs. biofilm communities, and the disruptive nature of sediment extraction to perform laboratory experiments and analyses. To overcome some of these challenges, it is crucial to develop and implement novel tools and approaches, including mVC monitoring and analytical chemistry approaches described herein coupled with microfluidic experiments. By utilizing these approaches in the study of NOM biotransformation in the terrestrial subsurface capillary fringe, we will be able to move toward closing a notable gap in our understanding of the terrestrial carbon cycle.

## Author Contributions

SG-D, KC, and RC contributed to the conception and outline of this review. SG-D, KC, SC, XW, AB, NB, and RC contributed substantially to drafting and revising the content of this work. All authors contributed to the article and approved the submitted version.

## Funding

This work was supported by ENIGMA – Ecosystems and Networks Integrated with Genes and Molecular Assemblies (http://enigma.lbl.gov), a Science Focus Area Program at Lawrence Berkeley National Laboratory is based upon work supported by the U.S. Department of Energy, Office of Science, Office of Biological & Environmental Research under contract number DE-AC02-05CH11231 and the U.S. Department of Energy, Office of Science, Office of Biological and Environmental Research, Early Career Research Program to NB (#FP00005182).

## Conflict of Interest

The authors declare that the research was conducted in the absence of any commercial or financial relationships that could be construed as a potential conflict of interest.

## Publisher’s Note

All claims expressed in this article are solely those of the authors and do not necessarily represent those of their affiliated organizations, or those of the publisher, the editors and the reviewers. Any product that may be evaluated in this article, or claim that may be made by its manufacturer, is not guaranteed or endorsed by the publisher.
